# Impact of Maine’s Statewide Nutrition Policy on High School Food Environments

**Published:** 2010-12-15

**Authors:** Janet E. Whatley Blum, Christina M. Beaudoin, Liam M. O'Brien, Michele Polacsek, David E. Harris, Karen A. O'Rourke

**Affiliations:** Department of Exercise, Health, and Sport Sciences, University of Southern Maine; Grand Valley State University, Allendale, Michigan; Colby College, Waterville, Maine; University of New England, Biddeford, Maine. At the time of the study, Dr Polacsek was affiliated with the Maine-Harvard Prevention Research Center, Augusta, Maine; University of Southern Maine, Portland, Maine; University of New England, Portland, Maine

## Abstract

**Introduction:**

We assessed the effect on the food environments of public high schools of Maine's statewide nutrition policy (Chapter 51), which banned "foods of minimal nutritional value" (FMNV) in public high schools that participated in federally funded meal programs. We documented allowable exceptions to the policy and describe the school food environments.

**Methods:**

We mailed surveys to 89 high school food-service directors to assess availability pre–Chapter 51 and post–Chapter 51 of soda, other sugar-sweetened beverages, and junk food. Frequency data were tabulated pre–Chapter 51 and post–Chapter 51, and Fisher exact test was used to assess significance in changes. We conducted food and beverage inventories at 11 high schools.

**Results:**

The survey return rate was 61% (N = 54). Availability of soda in student vending significantly decreased pre–Chapter 51 versus post–Chapter 51 (*P* = .04). No significant changes were found for other sugar-sweetened beverages and junk food. Exceptions to Chapter 51 were permitted to staff (67%), to the public (86%), and in career and technical education programs (31%). Inventories in a subset of schools found no availability of soda for students, whereas other sugar-sweetened beverages and junk food were widely available in à la carte, vending machines, and school stores. Candy, considered a FMNV, was freely available. Soda advertisement on school grounds was common.

**Conclusions:**

Student vending choices improved after the implementation of Chapter 51; however, use of FMNV as the policy standard may be limiting, as availability of other sugar-sweetened beverages and junk food was pervasive. School environments were not necessarily supportive of the policy, as advertisement of soda was common and some FMNV were available. Furthermore, local exceptions to Chapter 51 likely reduced the overall effect of the policy.

## Introduction

School food environments have been identified as a factor in children's nutrition (1-3). Schools participating in US Department of Agriculture (USDA) federal meal programs (ie, the National School Lunch Program and the School Breakfast Program) are required to follow nutrition standards for federal meals and competitive foods (4). USDA defines competitive foods as any foods sold at school outside of and in competition with the federal meal programs (4). Competitive foods that are considered "foods of minimal nutritional value" (FMNV) are restricted from sale in the cafeteria during breakfast and lunch (4). However, FMNV can be sold at any time, anywhere else on school grounds, including in vending machines, school stores, and snack bars. FMNV are defined as providing less than 5% of the recommended daily intake for each of 8 specified nutrients (protein, vitamin A, vitamin C, niacin, riboflavin, thiamin, calcium, and iron) per serving for artificially sweetened foods or per 100 calories and per serving for all other foods (4). Federal regulation has no authority over competitive foods with high fat or high sugar content if they meet the FMNV threshold (eg, chips, sugar-sweetened beverages). The availability of food with poor nutrient quality in competitive food venues may contribute to a poorer dietary intake and increase the risk for obesity in middle and high school students (5-8). It has been reported that school food environments may become less healthful as students move from elementary to high school, where vending programs and à la carte programs are more prevalent (9).

Recent efforts to improve school nutrition regulations include the Child Nutrition and WIC Reauthorization Act of 2004, which required schools to establish a wellness policy that includes nutrition guidelines for all foods at schools (10). To augment the reauthorization act, the Institute of Medicine established nutrition standards (eg, decreasing fat and added sugar) for foods and beverages offered outside of federal meal programs (2). A range of school nutrition standards have also been legislated or regulated in states during the last 5 years (11). Literature supports school-based food policy change. However, little is known about the effectiveness of policy changes on school food environments (8,12). A study of 20 Minnesota high schools found that school food policies that decreased access to foods high in fats and sugars were associated with less frequent purchase of these items in school by students (13). Policies associated with less frequent purchase of high-fat and high-sugar items included having a closed campus during lunch and limited hours of access to beverage vending machines (13). Another study that implemented low-fat, low-sugar nutrition guidelines to make healthier snacks and beverages available in several Maine high schools showed that guidelines were more easily achieved in vending programs than in à la carte programs (14).

One of the first statewide nutrition policies in the United States (Chapter 51) was implemented by the Maine Department of Education effective July 2005 (15). For many years, Maine had extended the USDA restriction of the sale of FMNV beyond breakfast and lunch to the entire school day. However, before and after school, most high schools had vending machines or other venues where students could purchase soda and candy. Chapter 51 further extended the USDA restriction of selling FMNV to any time on school grounds in any public school that participated in the federal meal programs (15). Therefore, FMNV were banned from being sold in vending machines, school stores, and club sales at any time. However, Chapter 51 did allow local school boards to make exceptions to the policy and allow FMNV to be sold to school staff, to the public at events held on school property, and in the instructional career and technical programs (15).

We examined the effect of Chapter 51 on high school food environments. Our primary goal was to examine the availability of soda in food venues before and after the implementation of Chapter 51. A secondary goal was to further describe high school food environments by examining the availability of other sugar-sweetened beverages (eg, sports drinks, fruit drinks) and junk food (eg, cookies, cakes, salty snacks not low in fat) in food venues before and after the implementation of Chapter 51, documenting local school board exceptions to Chapter 51 (eg, sale of soda to teachers) and taking an inventory of food venues and soda advertisements in a subset of high schools.

## Methods

### Study design

Maine has 98 high schools, and we identified 89 of them that participated in federal meal programs. We mailed surveys to the food-service directors of these schools to collect data on the availability pre–Chapter 51 and post–Chapter 51 of soda, other sugar-sweetened beverages, and junk food. We also collected data on local school board exceptions to Chapter 51 and current soda advertisement data. Our survey tool was adapted from Probart et al (16). From these 89 high schools, we randomly selected 11 high schools for additional inventory and observational data collection. We mailed surveys and inventoried schools in the fall (September and October) of 2006, 1 year after Chapter 51 was implemented. This study was approved by the institutional review board of the University of Southern Maine.

We report results on the availability of soda, other sugar-sweetened beverages (ie, sport drinks, fruit drinks, and water with added sugar), and junk food (ie, chocolate candy, nonchocolate candy, cookies, crackers, cakes, pastries, bars, or other baked goods not low in fat, salty snacks not low in fat, and ice cream not low in fat) for sale in à la carte programs, in vending machines, in school stores, and at club sales. We adapted response choices to gather information (yes or no) retrospectively, pre–Chapter 51 (2004-2005 school year) and post–Chapter 51 (2006-2007 school year) (Appendix).

To determine allowable exceptions to Chapter 51, we asked, "Are there exceptions to the regulations of Chapter 51 for the sale of foods and beverages outside the total food service program 1) to staff, 2) to the public at community events sponsored by the school or held on school property, 3) to the public at community events held on school property in accordance with the school board's facilities policy, and 4) in instructional career and technical education programs?" Responses options were yes, no, or "don't know." We also included questions about current soda advertisements in the cafeteria, on vending machines, in school buildings, or on school grounds.

We mailed the survey and included a cover letter, anonymous survey consent, a $10 check, and a stamped return envelope. We mailed a postcard 1 to 2 weeks after the initial mailing to thank recipients for completing the survey or remind them to return the survey, and we mailed a second survey to nonrespondents 1 to 2 weeks after the postcard was sent (16). Only food-service directors who were employed in their position before and after the implementation of Chapter 51 were asked to complete the survey.

### Inventory and observational data collection

To meet the requirements of the funding organization, we inventoried only high schools in which at least 30% of the students were eligible for free or reduced-cost school lunches. We randomly selected 1 school from each of Maine's 16 counties. Three counties had no eligible schools, and the schools in 2 counties did not respond to letters of inquiry, which left a study group of 11 high schools. A team of 2 researchers visited each of these 11 schools during the fall of 2006 (October and November) to collect data to describe the food environments. We used a previously developed inventory tool to assess food and beverages in à la carte programs, vending machines, and school stores (17). Club sales were not inventoried.

Inventories assessed sugar-sweetened beverages (sport drinks, fruit drinks, and water with added sugar), non–sugar-sweetened beverages (water, water with artificial sweetener, 100% fruit juice, low-fat milk, and reduced-calorie sport drinks), junk food (chocolate candy, nonchocolate candy, salty snacks not low in fat, ice cream not low in fat, and cookies, crackers, cakes, pastries, bars, or other baked goods not low in fat), and non–junk food (low-fat salty snacks, low-fat ice cream and low-fat cookies, crackers, cakes, pastries, or other baked goods). We also noted soda consumption by students and the availability of candy considered a FMNV.

### Statistical analysis

We used completed food-service surveys to assess pre–Chapter 51 (2004-2005 school year) and post–Chapter 51 (2006-2007 school year) availability of soda, other sugar-sweetened beverages, and junk food in à la carte programs, in vending machines, in school stores, and at club sales. We assessed significance in the number of schools offering soda, other sugar-sweetened beverages, and junk food pre–Chapter 51 and post–Chapter 51 by using the Fisher exact test. Significance was set at *P* < .05. We used frequency data to tabulate exceptions to Chapter 51 and tabulated location of current soda advertisements from survey data. We also used frequency data to calculate inventory data from the subset of observed schools. Statistical analyses were conducted by using Stata SE version 10 (StataCorp LP, College Station, Texas).

## Results

### Food-service directors' survey

Two food-service directors reported they did not hold their position pre–Chapter 51 and therefore did not complete the survey. Fifty-four school food-service directors returned completed surveys, a response rate of 61%. Response rates for questions varied because some schools had no instructional career and technical education programs, school stores, or club sales, and because of missing data.

The survey revealed that the number of schools with soda available in student vending programs decreased significantly after the implementation of Chapter 51 (Fisher exact test,* P* = .04). No other significant changes were found for any other food or beverage item pre–Chapter 51 versus post–Chapter 51 in à la carte programs, student vending, in school stores, or at club sales (Table 1). Soda advertisements were reported to exist in the cafeteria area in 2 schools, on vending machines in 10 schools, and outside the building (ie, score boards) in 13 schools.

Local school board exceptions to Chapter 51 were reported in 35 schools to staff, in 45 schools to the public at community events sponsored by the school, in 45 schools to the public at community events held on school property in accordance with the school board's facilities policy, and in 14 instructional career and technical education programs.

### Inventories and observational data

Inventories from the 11 schools showed no soda in any student food venue (ie, à la carte, student vending, and school stores). However, in 4 schools, students were observed drinking soda. Inventories of staff vending machines revealed that soda was sold in 8 schools, and additional observation revealed 4 schools that had staff refrigerators stocked with soda.

Other sugar-sweetened beverages were sold in student vending programs in 10 schools, 6 staff vending programs, and 3 à la carte programs. Junk food was sold in student vending programs in 8 schools, 7 staff vending programs, and 3 à la carte programs. Our inventories of each type of item sold in student vending programs revealed that one-third of the beverage items sold were sugar-sweetened, and about one-half of the snack items sold were junk food (Table 2).

Four schools had stores that sold food or beverages or both. None of these stores sold soda; however, 3 school stores sold other sugar-sweetened beverages, and all 4 school stores sold junk food. Candy meeting the FMNV standard (eg, peanut M&Ms, Skittles, Starburst) was sold in all school stores inventoried. Anecdotally, school store managers noted they "searched" for candy that met the FMNV standard to sell. Managers perceived there would be loss of revenue if all candy were eliminated from school stores, but no manager could provide revenue data to support this perception. In about one-half of the schools we observed, bowls or baskets of candy that were FMNV (eg, candy corn, hard candy) were made readily available to students (ie, on the desks of principals, secretaries, or guidance counselors).

Observational data revealed that 10 schools had soda advertisements, mainly on indoor and outdoor scoreboards, and that 1 school advertised candy.

## Discussion

We found a significant reduction in the number of schools that sold soda, a food of minimal nutritional value, in high school student vending programs after the implementation of Maine's statewide nutrition policy, Chapter 51. Because Maine public schools already extended the USDA's regulation of the sale of FMNV to students for the entire school day, Chapter 51 merely decreased the sale of soda to students before and after school and did not result in the elimination of FMNV at all times on school grounds. Moreover, because Chapter 51 does not restrict students from bringing FMNV to school, students we observed consuming soda during the school day most likely brought the soda with them. Home may be the primary nonschool source and location for consumption of soda and other sugar-sweetened beverages for both boys and girls aged 14 to 17 years (18,19). Furthermore, Wang et al found that schools may not be a significant source of sugar-sweetened beverages, including soda (1%-7%), and overall consumption of these types of beverages at school is low (approximately 7%-15% of total consumption) for children and adolescents (19). Recent data from Maine high schools also suggest that schools may be limited in their role in reducing the consumption of sugar-sweetened beverages by students when the nutrition policy implemented only eliminates 1 type of sugar-sweetened beverage (20). Therefore, only small changes in students' consumption of soda and other sugar-sweetened beverages as a result of school policy changes should be expected. Nevertheless, consumption of soda and other sugar-sweetened beverages has been shown to be a potential contributor to weight gain in youth (21-23).

Many of the schools we studied allowed exceptions to Chapter 51; more than half allowed the sale of FMNV to staff and more than three-fourths allowed the sale to the public on school grounds. A study of 56 California high schools also reported differences in adherence to state-mandated nutrition standards regarding the percentage of beverage or food items that met the standard (24). Other studies suggest that implementation and enforcement of state-level or district-level nutrition policies vary among schools (14,25,26).

Because Maine's Chapter 51 used the USDA's FMNV standard (4), items with poor nutrient content, such as sugar-sweetened beverages or potato chips, are not eliminated from competitive food venues. The USDA's FMNV standard has been described as "weak, outdated, and arbitrary" (27). Efforts are under way to improve and update the federal regulations for school competitive food venues (2). Stronger food-service policies — such as the Texas Public School Nutrition Policy, which restricts portion sizes of high-fat and high-sugar snacks and sweetened beverages, limits fat content of foods, and limits frequency of serving high-fat vegetables — show promising results for improving the diets of middle school students (28,29). Recent data also suggest that the presence of additional school food practices, such as the use of food as a reward or allowing food and beverage consumption in the classroom, are associated with higher body mass index in middle school students (30).

We found that candy was readily available in schools, particularly in school stores, and that store managers were concerned that its elimination could negatively affect school store revenue. However, a recent review by Wharton et al (31) suggests that offering healthier foods or eliminating foods and beverages with poor nutrient quality in competitive food venues (ie, cafeteria à la carte and vending programs) does not negatively affect school revenues. Further examination of revenue data when changes in offerings are made in food venues like school stores is warranted.

We found that school soda advertising was less than supportive of the policy, especially with reference to scoreboards. This result mirrors a recent national survey that found 67% of schools had advertising for foods high in fat or sugar or both (32). Food advertising affects children's food choices, food purchase requests, diets, and health (33), and signage on school campuses affects students' food selection at school (34). Because no soda was available to students during the school day, the soda advertisements on scoreboards likely would have the greatest effect on beverage choices during events held on school grounds open to the public as well as student choice of beverage outside the school day. At the time of the observation, the soda industry had promised to eliminate soda advertisements from scoreboards in the state of Maine. Therefore, our data were used to aid in the passage of legislation that now bans brand-specific advertisement of junk food on school grounds, effective September 2007 (35).

Our study has several limitations. The study sample was small and included only Maine public high schools, so our results may not be generalizable. Approximately 40% of food-service directors did not respond to the survey, so our results may be biased. Collection of data from food-service directors was cross-sectional and required directors to recall availability of foods and beverages approximately 1 year prior. Furthermore, food-service directors were asked about availability of foods and beverages in all food venues in the high schools and may not have had accurate knowledge of availability in food venues that were not under their direct supervision.

Maine's statewide nutrition policy improved some student vending choices. However, the overall effect of Chapter 51 was most likely reduced because the majority of schools took advantage of local school board authority to allow exceptions. Further understanding of why most schools did not fully implement Chapter 51 may be needed so future policies address schools' concerns. Maine high school environments were not necessarily supportive of Chapter 51, and it may take stronger and more explicit school nutrition policies that are monitored and enforced to positively affect school food environments. Furthermore, the use of USDA's FMNV standard as the basis for a nutrition policy in school may be limiting. Our results support the need to strengthen the USDA's federal nutrition standards. Policies should cover a broader range of foods and beverages with poor nutrient quality that are available in competitive food venues so that substantial improvements in school food environments can be made (28).

## Figures and Tables

**Table 1:** Number of Maine Public High Schools (N = 54) With Availability of FMNV, Before (2004-2005 School Year) and After (2006-2007 School Year) Implementation of Chapter 51^a^

Food Item	Student Vending^b^		Cafeteria à la Carte^c^		School Stores^d^		Club Sales^e^	

	Before Chapter 51, n (%)	After Chapter 51, n (%)	Before Chapter 51, n (%)	After Chapter 51, n (%)	Before Chapter 51, n (%)	After Chapter 51, n (%)	Before Chapter 51, n (%)	After Chapter 51, n (%)
**Soda**	8 (17)	2 (4)^f^	2 (4)	1 (2)	3 (21)	1 (6)	2 (7)	0
**Other sugar-sweetened beverages**								
Sport drinks	35 (75)	30 (58)	23 (46)	19 (36)	5 (36)	4 (25)	5 (17)	4 (13)
Fruit drinks	17 (36)	13 (25)	20 (40)	15 (28)	2 (14)	1 (6)	2 (7)	3 (10)
Water with added sugar	11 (23)	14 (27)	13 (26)	12 (23)	3 (21)	3 (19)	2 (7)	2 (7)
**Junk food**								
Chocolate candy	4 (9)	2 (4)	2 (4)	0	5 (36)	2 (13)	7 (25)	4 (13)
Nonchocolate candy	4 (9)	3 (6)	3 (7)	0	5 (36)	3 (19)	7 (25)	3 (10)
Cookies, cakes, not low-fat	15 (33)	10 (20)	21 (46)	16 (33)	5 (36)	2 (13)	7 (25)	5 (17)
Salty snacks, not low-fat	19 (42)	16 (32)	23 (50)	18 (37)	5 (36)	2 (13)	3 (11)	3 (10)
Ice cream, not low-fat	5 (11)	5 (10)	22 (48)	18 (37)	1 (7)	1 (6)	2 (7)	2 (7)

Abbreviation: FMNV, foods of minimal nutritional value.

a Chapter 51 is a statewide nutrition policy that was implemented in July 2005 by the Maine Department of Education and bans the sale of FMNV on school grounds at any time in any public school that participates in federally funded meal programs.

b Data reported by 47 schools pre– and 52 post–Chapter 51 for soda and other sugar-sweetened beverages, 45 schools pre– and 50 schools post–Chapter 51 for junk food.

c Data reported by 50 schools pre– and 53 post–Chapter 51 for soda and other sugar-sweetened beverages, 46 schools pre– and 49 schools post–Chapter 51 for junk food.

d Data reported by 14 schools pre– and 16 post–Chapter 51 for soda, other sugar-sweetened beverages, and junk food.

e Data reported by 29 schools pre– and 31 post–Chapter 51 for soda and other sugar-sweetened beverages, 28 schools pre– and 30 schools post–Chapter 51 for junk food.

f Significant difference between pre– and post–Chapter 51 soda availability calculated by using Fisher exact test, *P* = .04.

**Table 2:** Inventory of Student Vending Machine Items, 11 Public High Schools in Maine, Fall 2006

Observed School	Types of Vending Machine Items			

	Sugar-Sweetened Beverages^a^	Non–Sugar-Sweetened Beverages^b^	Junk Food^c^	Non–Junk Food^d^
1	0	46	16	24
2	22	164	7	28
3	35	53	0	0
4	0	98	0	0
5	32	60	13	8
6	0	196	38	36
7	40	83	0	0
8	0	55	1	3
9	131	49	13	26
10	110	190	34	20
11	157	150	27	8

a Sugar-sweetened beverages were sport drinks, fruit drinks, and water with added sugar.

b Non–sugar-sweetened beverages were water, water with artificial sweetener, 100% fruit juice, low-fat milk, and reduced-calorie sport drinks.

c Junk food was chocolate candy, nonchocolate candy, salty snacks not low in fat, ice cream not low in fat and cookies, crackers, cakes, pastries, bars, or other baked goods not low in fat.

d Non–junk food included low-fat salty snacks, low-fat ice cream and low-fat cookies, crackers, cakes, pastries, or other baked goods.

## Appendix. Sample Question From the Food-Service Directors' Survey, Maine Public High Schools (N = 54), Fall 2006

Question: Which of the following types of beverages and snacks are offered to students through **vending machines** at the high school? (Please check all that apply)

**Table:**

Item	2004-2005	2006-2007
**Beverages**		
Whole milk, plain or flavored		
2% Milk, plain or flavored		
1% Milk, plain or flavored		
Skim milk, plain or flavored		
Regular soda (full calorie) (eg, Coke, Pepsi, Sprite)		
Diet soda		
Sport drinks (full calorie) (eg, Powerade, Gatorade)		
Sport drinks (reduced calorie) (eg, Powerade options)		
Bottled water (plain) (eg, Dasani, Aquafina)		
Bottled water (with added sugar) (eg, Propel)		
Bottled water with artificial sweeteners (eg, Fruit2O, Dasani Raspberry)		
100% Fruit juice		
100% Vegetable juice (eg, V8, tomato)		
Sweetened drinks (not 100% juice) (eg, lemonade, fruit punch, iced tea)		
Energy drinks (eg, Red Bull)		
Other:		
Do not know		
**Snacks**		
Chocolate candy		
Nonchocolate candy (eg, Skittles)		
Low-fat cookies, crackers, cakes, pastries, or other baked goods		
Cookies, crackers, cakes, pastries, or other baked goods not low in fat		
Salty snacks that are not low in fat (eg, regular potato chips)		
Salty snacks that are low in fat (eg, Baked Lays, pretzels)		
Ice cream or frozen yogurt that is not low in fat		
Low-fat or fat-free ice cream, frozen yogurt, or sherbet		
Low-fat or nonfat yogurt		
Fruits or vegetables		
Bread sticks, rolls, bagels, pita bread, or other bread products		
Other:		
Do not know		
